# Commentary: ED_50_ and ED_95_ of hypobaric ropivacaine during unilateral spinal anesthesia in older patients undergoing hip replacement surgery

**DOI:** 10.3389/fmed.2025.1724654

**Published:** 2026-01-20

**Authors:** Songfeng Li, Yalin Tian, Shanhuai Tian, Tingdan Tan, Shaojin Bu

**Affiliations:** 1 Department of Anesthesiology, Fengdu People's Hospital, Chongqing, China; 2 Department of Emergency, Fengdu People's Hospital, Chongqing, China; 3 Department of Pediatrics, Fengdu People's Hospital, Chongqing, China

**Keywords:** dose-response relationship, geriatric anesthesia, methodology, probit analysis, ropivacaine, spinal anesthesia

## Introduction

Lin et al. (1) provided crucial clinical insights into dosage optimization for unilateral spinal anesthesia in older adults, a demographic vulnerable to anesthetic complications. Although the authors utilized Dixon's sequential design, a methodologically apt approach for estimating median effective doses, a thorough examination of the reported dose-response parameters reveals a significant pharmacological inconsistency that warrants discussion for the accurate interpretation of the findings.

## A pharmacological impossibility: ED_95_ < ED_50_

In Table 2 and the Results section, the authors reported an ED_50_ of 11.13 mg (95% CI: 10.85–11.42 mg) and an ED_95_ of 10.30 mg (95% CI: 9.04–10.65 mg). This result contradicts the established pharmacological principles. By definition, within any monotonic dose-response framework (including Probit and Logit regression models), the dose needed to elicit a response in 95% of the population (ED_95_) must be greater than that for 50% (ED_50_) (2, 3). The reported inversion of these values is mathematically unexpected and may suggest an underlying issue in the model specification or interpretation.

## Methodological considerations: evidence of a coding artifact

A detailed examination of these figures reveals the source of this inconsistency. Figure 3 presents a dose-response curve labeled “Incidence of Negative Reactions (%),” which descends from 100% to 0% with increasing doses. This indicates that the authors modeled the probability of a negative response (failure to achieve a blockade) instead of a positive response (successful blockade). Hence, the reported “ED_95_” mathematically represents the dose at which 95% of patients do not achieve surgical anesthesia, while “ED_50_” reflects the dose for 50% failure. This misinterpretation inevitably inverts the expected relationship between the **two** values.

Additionally, the sequential design (Figure 2) constrains data points within a narrow range (10.0–12.0 mg) with alternating outcomes, typical of the up-down method focused on estimating ED_50_. This limited sampling can exacerbate the instability of extreme quantile estimates, such as ED_95_, when combined with a mis-specified model outcome.

## Clinical implications and the path to clarification

Accurate determination of ED_95_ is vital for patient safety in elderly individuals, who often exhibit reduced physiological reserve and altered pharmacodynamics. Published values may misguide the clinical dosing strategies. A reevaluation of the probit model specification is recommended, particularly with regard to the definition of the response variable. A re-analysis defining “success” as achieving surgical anesthesia should restore a monotonically increasing dose-response relationship and yield pharmacologically plausible values, where ED_95_ > ED_50_.

In the spirit of transparency and reproducibility, we included the complete statistical code used for our methodological verification as Supplementary material. This demonstrates the impact of response variable coding on the model output in the probit regression framework.

## Discussion

This commentary elucidates a critical yet often overlooked methodological pitfall in dose-response analysis: the accurate definition of the binary outcome variable in regression models. The inversion of the ED_95_ and ED_50_ values reported by Lin et al. stems specifically from modeling the probability of block failure rather than success. It is crucial to emphasize that this critique pertains to a methodological artifact in data analysis, not to the fundamental concept of the study or the integrity of the collected data. The original observations retain value and, if reanalyzed with the correct outcome specifications, are likely to yield pharmacologically plausible and clinically useful parameters.

Our analysis reinforces a fundamental tenet of pharmacodynamic research: meticulous verification of outcome coding is a prerequisite for the biological plausibility. This case serves as a salient reminder for researchers to rigorously validate the model protocols when employing binary outcomes. Beyond this specific error, our commentary highlights broader methodological considerations for dose-finding studies, particularly in vulnerable populations. Studies aiming to estimate extreme quantiles (ED_95_) must explicitly acknowledge the inherent limitations of sequential designs, such as Dixon's up-and-down method, which is optimized for estimating the median (ED_50_) and often yields unstable estimates at the tails of the distribution.

To enhance methodological rigor in future research, we recommend: (1) adopting clear, pre-specified definitions for “success” and “failure” outcomes prior to model fitting; (2) supplementing sequential design data with larger, confirmatory cohorts, where feasible, to improve the stability of extreme quantile estimates; and (3) considering more efficient contemporary trial designs that can jointly model multiple endpoints and adaptively allocate patients based on accumulating data, thereby providing more robust dose-response characterization (4, 5).

In conclusion, a correctly specified model will not only resolve the presented pharmacological impossibility but also significantly enhance the clinical utility and reliability of the authors' study. This discussion underscores the importance of methodological transparency and adherence to pharmacometric principles, aiming to contribute to the advancement of rigorous and reproducible dose-response research in anesthesiology.
