# Supplementary material for: Commentary: ED50 and ED95 of hypobaric ropivacaine during unilateral spinal anesthesia in older patients undergoing hip replacement surgery
Source: Front Med (Lausanne). 2026 Jan 20;12:1724654. doi: 10.3389/fmed.2025.1724654 (PMC12864411; doi:10.3389/fmed.2025.1724654)
Supplement: Supplementary file 1 [file Table_1.docx]

import pandas as pd

import numpy as np

import statsmodels.api as sm

import matplotlib.pyplot as plt

# 数据

data = {

"dose": [10,10.5,11,11.5,11,11.5,12,11.5,12,11.5,11,11.5,11,11.5,12,11.5,

11,10.5,11,10.5,11,11.5,11,11.5,11,10.5,10,10.5,11,10.5,11,11.5,

11,11.5,11,10.5,11,11.5,11,10.5],

"positive": [0,0,0,1,0,0,1,0,1,1,0,1,0,0,1,1,1,0,1,0,0,1,0,1,1,1,0,0,1,0,0,1,0,1,1,0,0,1,1,0]

}

df = pd.DataFrame(data)

df = sm.add_constant(df["dose"])

# 拟合 Probit 模型

model = sm.Probit(df["positive"], df[["const","dose"]])

result = model.fit()

print(result.summary())

# 提取参数

b0, b1 = result.params

z95 = 1.645

ED50 = -b0/b1

ED95 = (z95 - b0)/b1

# Delta method计算标准误

cov = result.cov_params()

var_b0, var_b1, cov_b0b1 = cov.iloc[0,0], cov.iloc[1,1], cov.iloc[0,1]

se_ED50 = np.sqrt((1/b1)**2*var_b0 + (b0/b1**2)**2*var_b1 - 2*b0/(b1**3)*cov_b0b1)

se_ED95 = np.sqrt((1/b1)**2*var_b0 + ((z95-b0)/b1**2)**2*var_b1 - 2*(z95-b0)/(b1**3)*cov_b0b1)

ED50_CI = (ED50 - 1.96*se_ED50, ED50 + 1.96*se_ED50)

ED95_CI = (ED95 - 1.96*se_ED95, ED95 + 1.96*se_ED95)

print(f"ED50 = {ED50:.2f} mg (95% CI: {ED50_CI[0]:.2f}–{ED50_CI[1]:.2f})")

print(f"ED95 = {ED95:.2f} mg (95% CI: {ED95_CI[0]:.2f}–{ED95_CI[1]:.2f})")

# 绘制剂量–反应曲线

x = np.linspace(9.5, 12.5, 100)

y = sm.distributions.norm.cdf(b0 + b1*x)

plt.scatter(df["dose"], df["positive"], color='blue', alpha=0.6, label='Observed')

plt.plot(x, y, color='red', label='Probit fit')

plt.axvline(ED50, color='green', linestyle='--', label=f'ED50={ED50:.2f}')

plt.axvline(ED95, color='orange', linestyle='--', label=f'ED95={ED95:.2f}')

plt.xlabel("Ropivacaine dose (mg)")

plt.ylabel("Probability of positive response")

plt.legend()

plt.show()
